# A Novel and Practical Method of Performing Atrioventricular Nodal Ablation via a Superior Approach in Patients with Refractory Atrial Fibrillation Undergoing Cardiac Resynchronization Device Implantation

**DOI:** 10.19102/icrm.2019.101201

**Published:** 2019-12-15

**Authors:** Helbert Acosta, Lina M. Viafara, Nayyab Hanif, Steven Acosta, Manasa Pagadala, Byron Acosta, Shravya Pothula, Courtney Peckosh, Julie Bear, Sergio Alzate-Ricaurte, Humberto Ballesteros, Angela De Las Salas, Toni Martin, Matthew Doepke

**Affiliations:** ^1^Trinity Medical Center, Rock Island, IL, USA; ^2^Cardiovascular Medicine, P.C., Moline, IL, USA; ^3^Abbott Laboratories, Chicago, IL, USA

**Keywords:** Ablation, atrial Fibrillation, atrioventricular node, CRT, superior approach

## Abstract

Atrioventricular node (AVN) ablation is a strategy to manage patients with drug-refractory atrial fibrillation (AF) and heart failure in whom cardiac resynchronization therapy (CRT) device implantation has been prescribed. This study describes a practical method to perform these two procedures using the same surgical site. Twenty-seven patients were indicated for AVN ablation and concurrent CRT device implantation while presenting with AF and rapid ventricular response (RVR) refractory to medical therapy. After placement of the right and left ventricular leads, a third puncture was made in the axillary vein to obtain access to perform the ablation. After hand-injecting contrast media through a RAMP™ (Abbott Laboratories, Chicago, IL, USA) sheath positioned in the right atrial cavity, the anatomical area corresponding to the AVN was identified using fluoroscopy cine runs obtained in the right anterior oblique and left anterior oblique projections. The adequate site for ablation was confirmed by the bipolar recording of a His-bundle deflection at the tip of the ablation catheter. Radiofrequency energy was delivered to achieve complete heart block. Subsequently, device implant was completed. The method was successfully applied in 27 consecutive cases, achieving permanent complete heart block in all patients. The mean radiofrequency time to achieve heart block was 110 seconds ± 43 seconds. The average procedural time including AVN ablation and device implant was 87 minutes ± 21 minutes. The images obtained with the hand-injected contrast media provided adequate information to readily identify the anatomical area corresponding to the AVN with 100% accuracy. This study suggests that ablation of the AVN can be safely and effectively accomplished via a superior approach in patients undergoing a CRT device implant.

## Introduction

Atrial fibrillation (AF) is the most common sustained cardiac arrhythmia and is associated with significant morbidity and mortality.^1,2^ Despite recent advances in pharmacological and nonpharmacological therapies for the management of AF, there remains a population of patients in whom these contemporary therapies are inadequate. Atrioventricular (AV) node (AVN) ablation combined with pacemaker implantation has emerged as an effective treatment strategy usually offered to patients with symptomatic AF with rapid ventricular response (RVR). Patients in this category are considered nonresponsive to (or cannot tolerate) AVN blocker agents, are of an advanced age or present with significant comorbidities, and are not considered candidates for invasive curative procedures such as catheter or surgical ablative therapies.^3,4^ Moreover, AF with RVR refractory to medical therapy has been shown to hinder the effectiveness of cardiac resynchronization therapy (CRT) due to a suboptimal percentage of successful biventricular pacing.^5–7^ In this population of patients, AVN ablation at the time of CRT device implant could be desirable.

Traditionally, AVN ablation is accomplished percutaneously via the femoral vein approach and could be performed before, during, or after device implantation. Although this approach is highly efficient for achieving complete heart block, it imposes the need for sterile preparation of an additional anatomical area, thus increasing the duration of the procedure and the chances of infection due to the potential for cross-contamination with the device implant site. These risks have led to a search for alternative methods to facilitate the performance of these two procedures in a more efficient manner.

Alternatively, AVN ablation via a superior approach (entering the superior vena cava via the subclavian or axillary vein) has been shown to be safe and feasible.^8^ However, two factors make this approach technically difficult and time-consuming, which are the lack of a multipolar catheter for His-bundle recording to help localize the anatomical area corresponding to the AVN and the challenges in catheter manipulation inherent in this unfamiliar approach. Therefore, we sought in the present study to develop a new method to facilitate the execution of these two procedures concomitantly by achieving complete heart block via a superior approach utilizing the same site as that of the device implant.

## Methods

This was a single-center prospective cohort study that enrolled consecutive patients undergoing AVN ablation and a concurrent CRT device implantation (relevant patients had a class I or II indication for a biventricular pacemaker or implantable cardioverter-defibrillator). All patients had AF (persistent or paroxysmal) with RVR refractory to medical therapy and were not considered candidates for curative catheter or surgical ablative therapies. All 27 study participants provided written informed consent prior to enrollment. Prospective data analysis, informed consent, and methods were reviewed and approved by the appropriate institutional review board and followed the Declaration of Helsinki ethical principles. Patients who met any of the following criteria were excluded: age younger than 18 years, advanced renal failure (creatinine ≥ 2.0 mg/dL), pregnancy, or the presence of congenital cardiac abnormalities.

**Figure 1:**
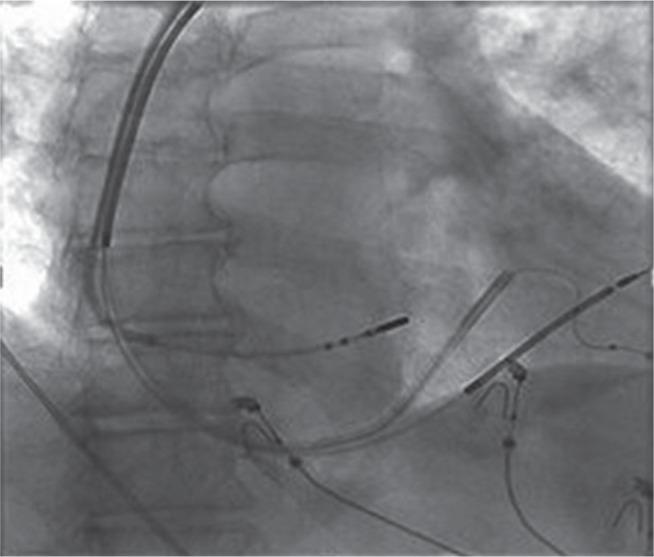
Right anterior oblique (20°) fluoroscopic image depicting the ablation catheter positioned at the successful ablation site.

**Figure 2:**
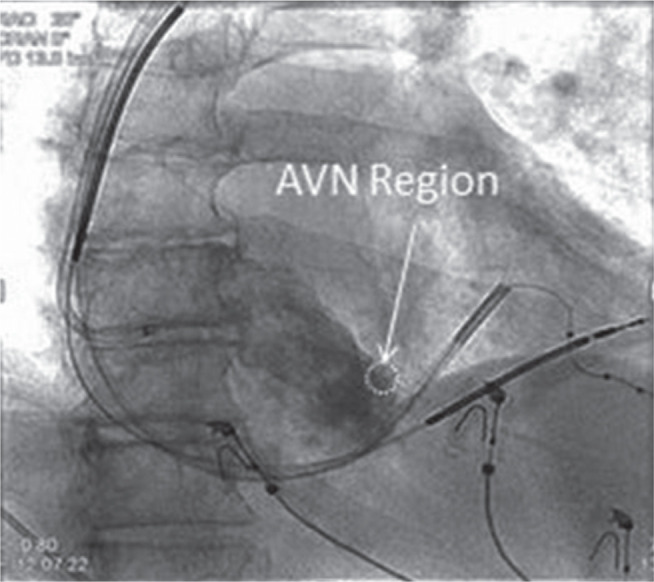
Right anterior oblique (20°) fluoroscopic image of the right atrium angiogram. White circle: location of the compact AVN.

AVN ablation was performed through the superior vena cava via the left or right axillary vein approach at the ipsilateral device implant site. After forming the pocket for the device, the right ventricular lead was inserted first and fixated at the midinterventricular septum. Subsequently, a left ventricular lead was placed in a (lateral) branch of the coronary sinus system using the standard “over-the-wire” technique. Next, a third separate puncture to the axillary vein was performed. An 8-French Fast-Cath™ Introducer RAMP™ series sheath (Abbott Laboratories, Chicago, IL, USA) was advanced over a 0.035-mm guidewire into the right atrial cavity. The tip of the sheath was then positioned inferiorly in the right atrial cavity with the tip oriented anteriorly and medially toward the intra-atrial septum. Subsequently, 3 mL to 5 mL of nonionic contrast medium was injected through the sheath using the hand-injection technique. Fluoroscopic images were obtained in the 20° to 30° right anterior oblique and in the 35° to 45° left anterior oblique projections. The information provided by these images was used to identify the anatomical location corresponding to the compact AVN **(Figure 1)**. An additional electrophysiologic catheter to record the His-bundle deflection directly was not used. Subsequently, a deflectable, bidirectional, 8-mm-tip ablation catheter (St. Jude Medical Safire™ TX Ablation Catheter; Abbott Laboratories, Chicago, IL, USA) was passed through the RAMP™ sheath (Abbott Laboratories, Chicago, IL, USA) into the right atrium and the tip was then oriented toward the preselected anatomical area corresponding to the compact AVN using a “frozen” image that was kept as a reference next to the live fluoroscopic screen **(Figure 2)**. The site corresponding to the AVN was confirmed by locating the bipolar recording of a small His potential and relatively large atrial potential on the distal electrodes of the ablation catheter. Subsequently, radiofrequency (RF) energy was delivered (power: 50–60 W; temperature: 50°C–60°C) to achieve complete AV block. Once complete AV block was achieved, additional RF lesions were delivered to consolidate the permanency of the block. After a waiting period of 20 minutes of persistent AV block, the ablation catheter and sheath were removed. The wire was either removed or retained depending on whether or not the patient was going to receive an atrial lead. Subsequently, the device implant was completed in a standard fashion. The total procedural time, total ablation time, and time from the initial ablation until the achievement of complete heart block were recorded in each case.

## Results

The superior AVN ablation technique was successfully applied in all the patients. A total of 27 patients (including 11 females) were enrolled, with a mean age of 72 years ± nine years. The general demographics, indications, and type of device implanted are shown in **Table 1**. The total procedural time, which included the ablation of the AVN plus device implant was 87 minutes ± 21 minutes. The duration of the RF ablation application was 110 seconds ± 43 seconds and the time from the delivery of the initial RF energy to the achievement of complete heart block was 33 seconds ± 25 seconds. Complete heart block was successfully achieved in all 27 patients; in 17 of 27 patients, complete AV block was achieved in less than 30 seconds of the delivery of the first RF energy. The His-bundle deflection was recorded at the tip of the ablation catheter upon its placement at the anatomical area selected using the fluoroscopy images. The images obtained during the hand-injection of contrast medium provided adequate information to identify the anatomical area corresponding to the AVN with 100% accuracy using a single shot. The procedure was performed via the left axillary vein in 26 patients and via the right axillary vein in one patient. No significant complications (eg, bleeding, hematoma, acute or late lead dislodgement, cardiac tamponade, pocket infection, stroke, effusion) occurred in any of the study participants.

All 27 patients in this study undergoing AVN ablation from a superior approach were ultimately discharged complication-free and without (right- or left-ventricle) lead dislodgement.

## Discussion

The technique described in this study proved to be an efficient, safe, and accurate protocol to achieve complete AV block via a superior approach in patients undergoing concomitant implantation of a CRT device. When a patient with AF presents refractory to medical management or has previously experienced intolerance to medications and has been ruled as ineligible for potentially curative ablative therapies, the “ablate and pace” approach is considered a well-accepted treatment strategy. In addition, AVN ablation has shown to be beneficial in patients with heart failure and AF managed with a CRT device because it enhances the degree of biventricular pacing.

Ablation of the AVN can be performed before, during, or after a CRT device is implanted. Although the femoral vein approach is the most widely utilized method to achieve complete AV block in these patients, it carries a series of technical and clinical disadvantages as follows:

1. The need for surgical preparation of an additional anatomical area, which increases the procedural time and the risk of cross-contamination and infection
2. Femoral venous access risks, including local bleeding, formation of arteriovenous fistulas, pseudoaneurysm, retroperitoneal bleeding, and infection.
3. If AVN ablation is performed prior to the implantation of the device, the patient could potentially be exposed to profound bradycardia and/or prolonged episodes of asystole should the backup pacing system fail to capture the ventricular chambers while the patient is waiting for the device to be implanted

These potential risks have led to a search for new methods or techniques to facilitate the concomitant performance of these two procedures in a more efficient and safe manner.

Issa et al. reported a series of 17 patients in which ablation of the AVN was successfully and safely accomplished via the left axillary or subclavian veins.^8^ The median number of RF applications required to achieve complete AV block was three (range: 1–10 applications). The procedural times and durations of RF energy delivery were not described. Additionally, the difficulties in reaching the AVN area without the use of a multielectrode catheter to facilitate the localization of the His bundle and the difficulties in manipulating an unsupported ablation catheter were not discussed.

In the traditional femoral approach, localization of the AVN area is mainly guided by the recording of the His-bundle deflection in a multielectrode catheter. Since the AVN itself cannot be recorded, the His-bundle deflection is used as an indirect marker of the AVN location. Usually, ablation is initiated when there is a small His-bundle deflection and a relatively large atrial electrogram recorded at the distal electrodes of the ablation catheter. However, the fact that the His-bundle potential could be recorded in a relatively large area of the AV junction made this technique relatively blind and somewhat imprecise in localizing the compact AVN area.

**Figure 3:**
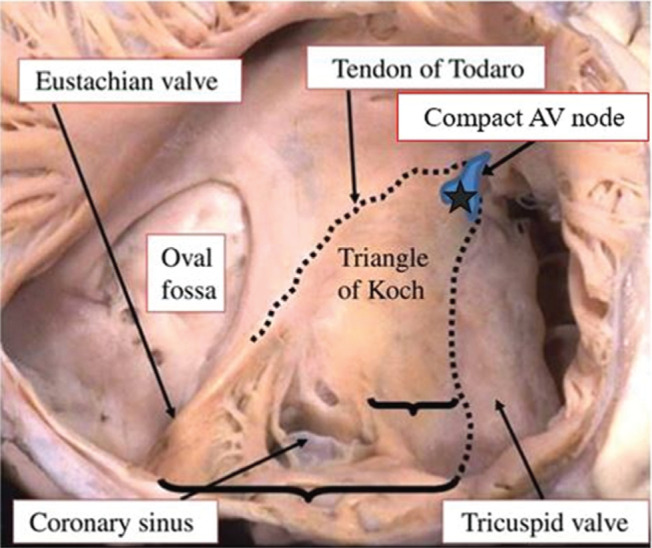
Photograph of human heart preparation showing the anatomical features of the triangle of Koch composed of the tricuspid valve anteriorly, tendon of Todaro posteriorly, and coronary sinus inferiorly. The compact AVN is located at the apex of the triangle of Koch (star). Obtained and modified with permission from Robert H. Anderson.

The technique described in our study represents a more precise radiographic/anatomical method to readily identify the anatomical area that corresponds to the compact AVN without the use of an additional multielectrode catheter to record the His-bundle potential. Anatomically, the compact AVN is situated near the apex of the triangle of Koch (which is constructed of the tricuspid annulus anteriorly, the tendon of Todaro posteriorly, and the coronary sinus ostium inferiorly) **(Figure 3)**. In the images obtained using a superior approach, a near-right angle is formed by the tricuspid annulus anteriorly and the superior border of the interatrial septum. The tip of the triangle of Koch is contained within this particular angle. Positioning the ablation catheter slightly (2–3 mm) inferior to the tip of this angle consistently provided a recording of the His-bundle potential and produced a prompt response, eliciting runs of junctional rhythm by the delivery of RF energy.

The relatively rapid localization of the His-bundle potential and the subsequent achievement of complete heart block with the applied technique is indicative of a high degree of precision and efficiency attributed to this method. The wide range of anatomical variations of the human heart could represent significant challenges in accomplishing AVN ablation. Our method would eliminate the partially blind search for the compact AVN area associated with a traditional approach. None of the patients in this study required a second ablation procedure promoted by a recurrence of conduction through the AVN.

Since AVN ablation and CRT device implantation can be performed via the same anatomical area, the need for sterile preparation of a second anatomical area is eliminated, thus shortening the procedural time and potentially reducing the chances for infection by avoiding cross-contamination between the two sites. A reduction in the procedural time could positively impact the cost efficiency of these procedures and enhance the workflow at the cardiac electrophysiology laboratory. Performing AVN ablation after the placement of the right and left ventricular leads could preclude the occurrence of profound bradycardia and/or episodes of prolonged asystole.

### Limitations

This is a nonrandomized observational study, which makes it difficult to define and compare characteristics with those of the traditional AVN ablation method. Although similar techniques have been utilized and observed at other centers, the data in this report were collected from a single center, thereby limiting their replicability. The number of patients (n = 27) in this report is also relatively low, limiting the potential clinical impact of the results and conclusions of our study. Larger randomized controlled studies would be expected to address some of these limitations.

## Conclusion

AVN ablation via a superior approach proved to be a safe, practical, efficient, and effective method to achieve complete AVN block in patients undergoing concurrent implantation of a CRT device for the management of patients with symptomatic atrial fibrillation with rapid ventricular response who are refractory to pharmacologic therapy.

## Figures and Tables

**Table 1:** Study Population Demographics, Indications, and Types of Devices Implanted

Age, (mean ± standard deviation)	72 ± 9 years
Gender	
Males	16 (52.3%)
Females	11 (40.7%)
Atrial fibrillation	
Persistent	26 (96.3%)
Paroxysmal	1 (3.7%)
Congenital cardiac abnormalities	0 (0%)
Device implanted	
Biventricular pacemaker	1 (3.7%)
Biventricular implantable cardioverter-defibrillator	26 (96.3%)
Medications being taken at the time of implant	
Beta-blockers	25 (92.6%)
Calcium channel blockers	1 (3.7%)
ACE inhibitors or ARBs	25 (92.6%)
Warfarin	23 (85.2%)
Aspirin	22 (81.5%)
Other anticoagulants	1 (3.7%)
Left ventricular ejection fraction	26% ± 11%

SD: standard deviation; ACE: angiotensin-converting enzyme; ARB: angiotensin II receptor blocker.
